# Excess fibroblast growth factor 23 in alcoholic osteomalacia is derived from the bone

**DOI:** 10.1093/jbmrpl/ziaf010

**Published:** 2025-01-16

**Authors:** Naoko Hidaka, Yuko Oyama, Minae Koga, Naoki Kondo, Yoichi Yasunaga, Taketoshi Shimakura, Noriaki Yamamoto, Hideaki E Takahashi, Yoichi Iwafuchi, So Watanabe, Soichiro Kimura, Yoshitomo Hoshino, Hajime Kato, Yuka Kinoshita, Hiroshi Kobayashi, Takeyuki Tanaka, Tetsuo Ushiku, Masaomi Nangaku, Sakae Tanaka, Noriko Makita, Taku Saito, Nobuaki Ito

**Affiliations:** Division of Nephrology and Endocrinology, The University of Tokyo Hospital, Tokyo 113-8655, Japan; Osteoporosis Center, The University of Tokyo Hospital, Tokyo 113-8655, Japan; Department of Nephrology, Saiseikai Niigata Kenoh Kikan Hospital, Niigata 955-0091, Japan; Division of Nephrology and Endocrinology, The University of Tokyo Hospital, Tokyo 113-8655, Japan; Osteoporosis Center, The University of Tokyo Hospital, Tokyo 113-8655, Japan; Division of Orthopedic Surgery, Department of Regenerative and Transplant Medicine, Graduate School of Medical and Dental Sciences, Niigata University, Niigata 951-8510, Japan; Department of Pathology, Graduate School of Medicine, The University of Tokyo, Tokyo 113-8655, Japan; Niigata Bone Science Institute, Niigata 950-3304, Japan; Niigata Bone Science Institute, Niigata 950-3304, Japan; Niigata Bone Science Institute, Niigata 950-3304, Japan; Department of Nephrology, Saiseikai Niigata Kenoh Kikan Hospital, Niigata 955-0091, Japan; Osteoporosis Center, The University of Tokyo Hospital, Tokyo 113-8655, Japan; Department of Geriatric Medicine, The University of Tokyo Hospital, Tokyo 113-8655, Japan; Division of Nephrology and Endocrinology, The University of Tokyo Hospital, Tokyo 113-8655, Japan; Osteoporosis Center, The University of Tokyo Hospital, Tokyo 113-8655, Japan; Division of Nephrology and Endocrinology, The University of Tokyo Hospital, Tokyo 113-8655, Japan; Osteoporosis Center, The University of Tokyo Hospital, Tokyo 113-8655, Japan; Division of Nephrology and Endocrinology, The University of Tokyo Hospital, Tokyo 113-8655, Japan; Osteoporosis Center, The University of Tokyo Hospital, Tokyo 113-8655, Japan; Division of Nephrology and Endocrinology, The University of Tokyo Hospital, Tokyo 113-8655, Japan; Osteoporosis Center, The University of Tokyo Hospital, Tokyo 113-8655, Japan; Osteoporosis Center, The University of Tokyo Hospital, Tokyo 113-8655, Japan; Department of Orthopedic Surgery, The University of Tokyo Hospital, Tokyo 113-8655, Japan; Orthopedic Surgery, Sensory and Motor System Medicine, Surgical Sciences, Graduate School of Medicine and Faculty of Medicine, The University of Tokyo, Tokyo 113-8655, Japan; Department of Pathology, Graduate School of Medicine, The University of Tokyo, Tokyo 113-8655, Japan; Division of Nephrology and Endocrinology, The University of Tokyo Hospital, Tokyo 113-8655, Japan; Department of Orthopedic Surgery, The University of Tokyo Hospital, Tokyo 113-8655, Japan; Division of Nephrology and Endocrinology, The University of Tokyo Hospital, Tokyo 113-8655, Japan; Osteoporosis Center, The University of Tokyo Hospital, Tokyo 113-8655, Japan; Osteoporosis Center, The University of Tokyo Hospital, Tokyo 113-8655, Japan; Department of Orthopedic Surgery, The University of Tokyo Hospital, Tokyo 113-8655, Japan; Osteoporosis Center, The University of Tokyo Hospital, Tokyo 113-8655, Japan; Division of Therapeutic Development for Intractable Bone Diseases, Graduate School of Medicine and Faculty of Medicine, The University of Tokyo, Tokyo 113-8655, Japan

**Keywords:** fibroblast growth factor 23, hypophosphatemia, osteomalacia, alcohol, immunohistochemistry, differential diagnosis

## Abstract

Excess fibroblast growth factor 23 (FGF23), a mature osteocyte-derived phosphaturic hormone, causes chronic hypophosphatemic osteomalacia in adults. This rare condition was recently reported in 2 alcoholic patients, with marked improvement upon cessation of alcohol consumption, suggesting a link between alcohol and FGF23-related hypophosphatemia within the highly limited cases. This study aimed to investigate whether the source of excess FGF23 in alcohol-induced FGF23-related hypophosphatemic osteomalacia is the bone or the other organs. To achieve this goal, an immunohistochemical approach for the bone obtained from a patient was employed. Initial attempts at quantifying FGF23 in the bone using conventional immunohistochemistry (IHC) faced issues in quantifiability and sensitivity for low FGF23 expression levels. Therefore, next-generation IHC with phosphor-integrated dots (PIDs) was applied, which enabled the quantification of FGF23 expression in the bone across a broad range. Preliminary analyses using IHC with PIDs on normal bone samples (*n* = 12) provided a reference level (154.5 PID particles per cell). IHC with PIDs quantified suppressed physiological FGF23 expression in the bone samples from 3 patients with tumor-induced osteomalacia, where FGF23 is oversecreted from a tumor (13.6 PID particles per cell). Subsequently, bone samples obtained from a 70-yr-old male with alcohol-induced FGF23-related hypophosphatemic osteomalacia were analyzed, showing a higher number of PID particles per cell (199.4 PID particles per cell) than the reference level. This study suggests that orthotopic, bone-derived FGF23 is implicated in alcohol-induced FGF23-related hypophosphatemic osteomalacia. Furthermore, the study also demonstrated that highly sensitive IHC with PIDs could aid in the differential diagnosis of FGF23-related hypophosphatemia of unknown origin. Specifically, a bone sample with a low number of PID particles per cell indicates an excess ectopic secretion of FGF23; a bone sample with a normal to high number of PID particles per cell indicates an excess orthotopic secretion of FGF23.

## Introduction

Chronic hypophosphatemia in adults results in muscle weakness and inhibition of bone mineralization, thus leading to features of osteomalacia, such as fragility fractures, bone pain, and dental complications.^1^^,^^2^ One of the causes of chronic hypophosphatemia in adults is the excess action of fibroblast growth factor 23 (FGF23), which is the key regulator of phosphate homeostasis. FGF23 promotes excess phosphate excretion into the urine by downregulating the expression of sodium-phosphate cotransporters (NaPi2a/NaPi2c) in the proximal tubules and reduces serum 1,25-dihydroxyvitamin D.^3^^,^^4^ Although chronic alcoholism is known to cause hypophosphatemia and bone fragility through mechanisms that are mainly related to vitamin D deficiency and damage to the renal tubules,^5^^,^^6^ we recently reported 2 alcoholic patients with hypophosphatemic osteomalacia, which was obviously induced by FGF23 oversecretion, and the condition was remedied by abstinence.^7^ Although FGF23 (in a physiological state) is specifically secreted from mature osteocytes or osteoblasts in bone, the source of excess FGF23 in these alcoholic patients is uncertain. A recent study demonstrated that hepatic FGF23 synthesis was induced in a murine model of chronic alcoholism with liver dysfunction.^8^ Furthermore, damaged livers were reported to ectopically synthesize FGF23. For example, pediatric patients with biliary atresia accompanied by hypophosphatemia were suggested to present with liver-derived FGF23 oversecretion, as determined by immunohistochemistry (IHC).^9^ Two patients with alcohol-induced FGF23-related hypophosphatemia (referred to as “alcoholic osteomalacia” hereafter) in previous reports also had mild liver dysfunction, which was improved by abstinence^7^; therefore, it is hypothesized that excess FGF23 occurred in these patients as a result of alcohol-induced liver damage.

In this study, to clarify the origin of excess FGF23 secretion in these patients, FGF23 in the bone sample was detected via IHC. When excess FGF23 is derived from bone, the expression of FGF23 should not be suppressed in the bone sample. Conversely, when excess FGF23 originates in the liver or organs other than bone, the expression of FGF23 should be physiologically suppressed in the bone. However, unfortunately, conventional IHC with 3,3′-diaminobenzidine (DAB) is not sensitive or quantitative enough to detect the suppression of FGF23. Thus, a novel method to quantify FGF23 expression over a wide range was developed by utilizing next-generation IHC with phosphor-integrated dots (PIDs). PID is a homogenous-sized nanoparticle with more than 100-fold greater bright fluorescence than other fluorescent molecules, such as quantum dots. Thus, IHC with PIDs provides significantly stronger signals that can be recognized at even the single-particle level.^10^ Furthermore, PIDs are photostable and automatically quantifiable using specialized software and analyzers; therefore, IHC with PIDs can be used to quantify the expression of targeted proteins over a wide dynamic range. IHC with PIDs has been introduced as a highly sensitive technique to quantitate the expression of targeted antigens, principally in cancer research.^11–15^ Although there have been no previous studies in which IHC with PIDs was applied to bone tissue, the quantification of the FGF23 expression level in the bone was expected to be substantiated with this method, thus enabling us to elucidate the pathophysiology of alcoholic osteomalacia.

This study described a newly identified patient with alcoholic osteomalacia in which hypophosphatemia recurred with alcohol recovery after remission with abstinence. Preliminary experiments were conducted to quantify the expression levels of FGF23 via IHC with PIDs in bone or other tissues obtained from patients with various conditions. Thereafter, the expression level of FGF23 in the bone biopsy sample obtained from the current alcoholic patient was quantitated via IHC with PIDs to determine the source of excess FGF23 under these conditions.

## Materials and methods

### Patients and preparation of tissue specimens

A bone biopsy sample from the described patient with alcoholic osteomalacia was obtained from the left iliac bone. Bone tissues from 14 patients who underwent implant arthroplasty at the Department of Orthopedic Surgery at our institution were collected for validation via IHC with PIDs to quantify FGF23 expression. These patients consisted of 12 patients with normal renal function (the standard group) and 2 patients who were undergoing hemodialysis (the CKD group). As the negative control, nonskeletal tissue (the nonskeletal group) was utilized, and the following 3 tissues that were surgically resected for the relevant diseases were obtained: the thyroid gland, lymph node, and stomach. In addition, among the patients with tumor-induced osteomalacia (TIO), normal bone tissues were excised concomitantly during wide excision of the phosphaturic mesenchymal tumors (PMTs) from 3 patients (the TIO group).

Each formalin-fixed specimen was embedded in paraffin, and the specimens obtained from the bone were decalcified with an ethylenediaminetetraacetic acid (EDTA)-based reagent for approximately 1 wk before paraffin embedding.^16^ The paraffin blocks were sliced into 4-μm-thick sections.

### FGF23 assay and systemic venous sampling of FGF23

FGF23 was assayed using an FGF23 chemiluminescent enzyme immunoassay (CLEIA) (Minaris Medical), which specifically detects intact FGF23 (the active form). FGF23 levels in FGF23-related hypophosphatemic patients were reported to be above 30 pg/mL with concomitant hypophosphatemia in this assay.^17–20^ Systemic venous sampling of FGF23 was performed for the described case of alcoholic osteomalacia. An experienced radiologist inserted a catheter through the right femoral vein, and blood samples were collected from 22 sites, including the bilateral internal jugular veins, bilateral brachial veins, bilateral subclavian veins, bilateral brachiocephalic veins, distal superior vena cava, proximal superior vena cava, proximal inferior vena cava, distal inferior vena cava, bilateral common iliac veins, bilateral internal iliac veins, bilateral external iliac veins, bilateral proximal femoral 7veins, and bilateral distal femoral veins (Figure S1A).

### Imaging tests to localize PMTs

In the described case of alcoholic osteomalacia, fluorine-18-fluorodeoxyglucose-positron emission tomography/computed tomography (^18^F-FDG PET/CT) and ^111^In-pentetreotide SPECT/CT were performed according to the latest standard protocols. The ^18^F-FDG PET/CT images obtained at 2 h after the injection of the nuclides were reviewed. For ^111^In-pentetreotide SPECT/CT, images at 4, 24, and 48 h after the injection of the nuclides were reviewed by specialized radiologists.

### Whole-genome sequence analysis

In the case of alcoholic osteomalacia, genomic DNA was extracted from 200 μL of peripheral blood using a DNA isolation kit (NucleoSpin Blood; Macherey-Nagel) according to the manufacturer’s instructions. Whole-genome sequencing was performed using the Illumina NovaSeq 6000 platform (Illumina, Inc.) following standard protocols provided by Novogene Co., Ltd. Using the UCSC human genome hg38 as the reference genome, SNPs, insertion-deletions (INDELs), copy number variations (CNVs), and structural variants (SVs) in 12 genes responsible for germline FGF23-related hypophosphatemia (*FGF23*, *PHEX*, *DMP1*, *ENPP1*, *FAM20C*, *FGFR1*, *GNAS*, *HRAS*, *KRAS*, *NF1*, *NRAS*, and *PTH1R*) and 3 genes with the potential to cause FGF23-related hypophosphatemia (*GALNT3*, *ANKH*, and *FGF1*) were comprehensively identified. The established pathogenic variants were screened, and homozygous intron variants with an allele frequency of less than 10% and variants at exon or exon-intron boundaries with an allele frequency of less than 1% were extracted.

### Bone biopsy

For the described case of alcoholic osteomalacia in an alcohol-drinking state, 3 cylindrical tissues at 5 and 8 mm in diameter were obtained from the left iliac crest under general anesthesia. Bone biopsy was performed after 1 mo of discontinuation of active vitamin D and inorganic phosphate supplements.

### Bone histomorphometric analysis

The specimens were prestained for 72 h using Villanueva bone stain. Following dehydration in an increasing concentration of alcohol, they were embedded in methyl methacrylate and maintained at 37 °C until they were fully polymerized. Embedded biopsy samples were subsequently sectioned on a microtome (Leica RM2255; Leica Inc.) at a thickness of 5 μm. Histomorphometric measurements were obtained with a system consisting of an epifluorescence microscope (Olympus BX50; Olympus America Inc.), a bone histomorphometry system, and a software program (Histometry RT Camera; System Supply, Co.), which were connected to an Epson computer (Shinshu Seiki Co., Ltd.).

### IHC with DAB

The paraffin sections were deparaffinized, and antigen retrieval was performed with 10 mM citrate buffer (pH: 6.0) for 40 min at 85 °C. Endogenous horseradish peroxidase activity was blocked with 3% hydrogen peroxide for 15 min at room temperature and then blocked with blocking buffer (BL001, Konica Minolta) for 15 min at room temperature**.** The specimens were incubated with 41.8 μg/mL monoclonal FGF23 antibody (FC1, a gift courtesy from Kyowa Kirin; RRID: AB_2924835) for 1 h at room temperature. After washing with PBS, the samples were incubated with a goat anti-mouse IgG secondary antibody (N-Histofine Simple Stain AP [MULTI], Nichirei Biosciences Inc.) for 30 min at 25 °C and subsequently treated with DAB chromogen (DAB Tablet, FUJIFILM Wako Pure Chemical Corporation). The samples were washed with PBS, fixed with 4% paraformaldehyde/PBS, stained with hematoxylin and automatically mounted in mounting medium using Tissue-Tek Glas g1 (Sakura Finetek Japan Co., Ltd.).

### IHC with PIDs

Deparaffinization, antigen retrieval, and blocking were performed as described above for the IHC method with DAB. The samples were incubated with 41.8 μg/mL monoclonal FGF23 antibody (FC1) overnight at 4 °C. After 3 sets of 5 min of washing with PBS, the samples were incubated with 2 μg/mL biotinylated rat anti-mouse IgG secondary antibody for 30 min at 25 °C. After washing with PBS, the samples were incubated with 0.06 a.u. of streptavidin-modified fluorescent nanoparticles (Konica Minolta) for 2 h at 25 °C. Fixation, staining with hematoxylin, and mounting procedures were the same as those described for IHC with DAB.

### Quantification of PID particles

The fluorescence signals of the PIDs were observed using a fluorescence microscope (BX53, Olympus) equipped with a UPLSAPO 40X2 (Olympus) objective lens and a charge-coupled device camera (DP-73, Olympus). For each patient, 5-10 microscopic fields from 1 to 3 slides, selected to contain a large number of nuclei, were analyzed. On the bone tissue slides, cortical bone was selected for analysis. The number of PID particles per cell was scored using the “nearest-neighbor method,” which was utilized to count the PID particles within 5.5 μm from a nuclear boundary and attributed 1 particle only to the nearest nucleus to avoid the duplication of the count; this procedure was performed using a PID analyzer (Konica Minolta).^12^ In a preliminary analysis, a distance of 5.5 μm in the “nearest-neighbor method” was validated as follows. The number of PID particles per cell obtained via the “nearest-neighbor method” at distances of 2.2, 5.5, and 8.8 μm from the nuclear boundary were compared with the true value obtained by manually counting the number of PID particles attributed to each cell. The PID particles per cell that were obtained using the distance of 5.5 μm in the “nearest-neighbor method” demonstrated the strongest correlation with the true value (*r* = 0.92, Figure S2). IHC with PIDs and its quantification using the PID analyzer has been commercially available until April 2024 as “Quanticell,” previously provided by Konica Minolta REALM Inc. The resumption and its schedule of this analytical service are currently undecided.

### Statistics

The number of PID particles per cell for a sample was calculated as the average number of PID particles per cell counted by the abovementioned “nearest-neighbor method” using a PID analyzer with the following formula: (all PID particles counted)/(the number of observed nuclei). The numbers of PID particles per cell in the CKD, nonskeletal, and TIO groups were compared with those in the standard group via the Mann–Whitney U test using R software (version 4.3.2). The statistical test was 2-sided, and differences were considered to be statistically significant at a *p* value <.05. All of the data in the tables are presented as the mean and standard deviation.

### Study approval

All of the procedures were performed following the ethical standards of the Declaration of Helsinki and were approved by the institutional ethical board of the University of Tokyo Hospital [Ref. G10042]. All of the patients provided written informed consent before enrollment in the study.

**Figure 1:**
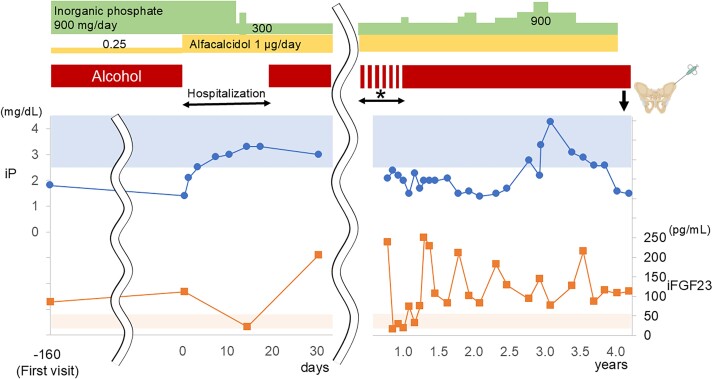
The clinical course of the patient with alcoholic osteomalacia. During admission to the hospital, complete abstinence was achieved. However, after discharge, the patient resumed alcohol intake, although he was able to stop drinking alcohol several times (a few days per time) within 1 yr (asterisk). The dose of inorganic phosphate supplementation (top block) was adjusted between 300 and 900 mg/day according to the level of serum inorganic phosphate. Bone samples were obtained at 4.1 yr after the first admission, when inorganic phosphate and alfacalcidol were discontinued for 1 mo and regular alcohol intake was continued. The reference ranges of serum inorganic phosphate (iP) and intact FGF23 (iFGF23) are shown in the top and bottom bands in graph area, respectively. The admission day was defined as “day 0.”

## Results

### Case description

A 70-yr-old man visited the orthopedic department in our hospital with painful swelling of the right dorsum of his foot, bilateral rib pain, and muscle weakness of his lower limbs. He had no history of fragility fractures or bone metabolic disease and drank 1000-2000 mL of spirits (*sake*, Japanese rice wine) with 15% alcohol (150-300 g of pure ethanol, 7.5-15.0 units) every day for more than 15 yr. An X-ray demonstrated fresh and old fractures of the right metatarsal bones. The laboratory data revealed hypophosphatemia (serum inorganic phosphate [iP]: 1.8 mg/dL), increased alkaline phosphatase (ALP [747 U/L]), and bone-specific ALP (BAP [173.9 μg/L]) with inappropriately high intact FGF23 (85 pg/mL, reference range under chronic hypophosphatemia: <30 pg/mL; Minaris Medical). Consistent with FGF23-related hypophosphatemia, the urinary excretion of phosphate was high (tubular maximum for phosphate reabsorption per glomerular filtration rate [TmP/GFR]: 1.2 mg/dL). Moreover, increased aspartate transaminase, alanine transaminase, and γ-glutamyltransferase levels indicated mild liver dysfunction due to habitual alcohol intake. ^99m^Tc-hydroxymethylene diphosphonate (HMDP) scintigraphy demonstrated multiple uptakes in the bilateral ribs, right ilium, left femoral neck, right distal femur, and bilateral feet (Figure S3A). These findings led to the diagnosis of FGF23-related hypophosphatemic osteomalacia. With no history of skeletal disease in his family members and an acquired form of his condition, TIO, which is the most prevalent cause of acquired FGF23-related hypophosphatemic osteomalacia, was initially diagnosed. He was admitted to our hospital and subjected to thorough examinations, including systemic venous sampling of FGF23, to locate the culprit tumor of the TIO. Neither ^111^In-pentetreotide SPECT/CT nor ^18^F-FDG PET/CT indicated any tumors (Figure S1B and C). On the day of admission (“day 0” in Figure 1), hypophosphatemia (iP: 1.4 mg/dL) with inappropriately high intact FGF23 was confirmed (intact FGF23: 111 pg/mL) (Table S1). However, the serum phosphate and intact FGF23 levels normalized within 2 wk when systemic venous sampling of FGF23 was conducted. Therefore, intact FGF23 levels at 22 sampling points in systemic venous sampling were similarly low (approximately 20 pg/mL) (Figure 1; Figure S1A). The spontaneous remission of FGF23-related hypophosphatemia after admission was similar to that observed in previous patients with alcoholic osteomalacia and was reversible by alcohol abstinence.^7^ Therefore, the serum phosphate and FGF23 levels were closely monitored after discharge. The clinical course and changes in laboratory data over approximately 4 yr are presented in Figure 1 and Table S1. Hypophosphatemia and an inappropriate increase in FGF23 levels recurred after the patient resumed drinking, which confirmed the diagnosis of alcoholic osteomalacia (Figure 1; Table S1). Supplementation with inorganic phosphate and active vitamin D remitted the symptoms of osteomalacia, which was confirmed by ^99m^Tc-HMDP scintigraphy after 4 mo (Figure S3B) and a decrease in ALP and BAP (Table S1). Despite the recommendation of abstinence, he continued drinking alcohol. Active vitamin D and inorganic phosphate supplementation were continued for 4 yr. Within the follow-up period, the liver fibrosis stage progressed from F1 to F3, as indicated by a 2-dimensional shear wave elastography value calculated via echography (8.0-15.1 kPa) (Table S1).^21^ To elucidate whether he had a genetic background for inherited forms of FGF23-related hypophosphatemia, whole-genome sequence analysis was conducted, and 12 genes responsible for FGF23-related hypophosphatemia and 3 genes with the potential for FGF23-related hypophosphatemia were investigated. However, no pathogenic or rare variants of unknown significance were found in these genes.

**Figure 2:**
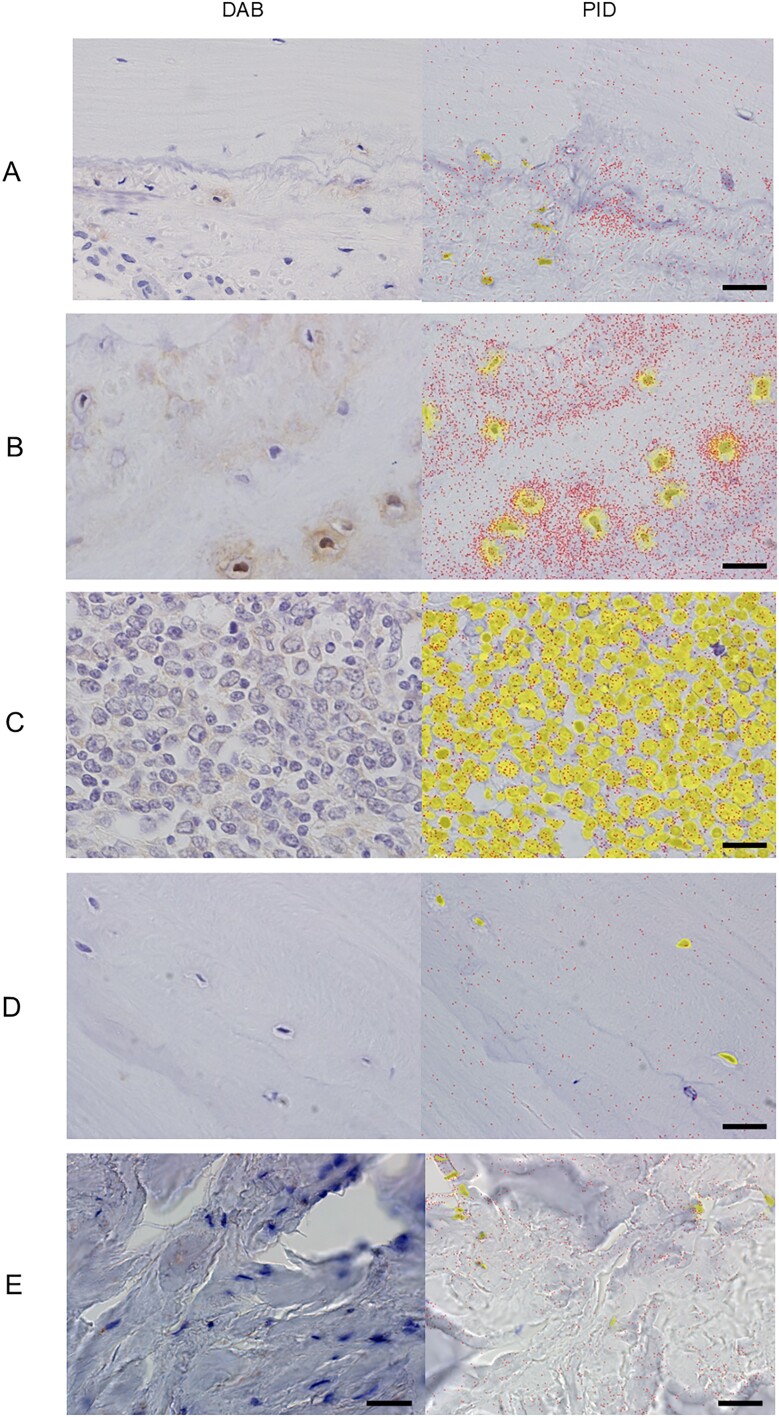
Representative images of immunostaining using DAB or PIDs for FGF23 in the following patients: (A) standard group, (B) CKD group, (C) nonskeletal group, (D) TIO group, and (E) the patient with alcoholic osteomalacia. In the images of immunostaining with PIDs, PID particles (dots) were counted for the observed cell nuclei (circled regions around cells) via the nearest-neighbor method using a PID analyzer. The scale bars (right bottom) were defined as 20 μm. Abbreviations: TIO, tumor-induced osteomalacia; PID, phosphor-integrated dot; DAB, 3,3′-diaminobenzidine.

**Table 1:** Characteristics and laboratory parameters of patients in which tissue samples were subjected to validation by IHC with PIDs for FGF23.

**Characteristics and laboratory parameters**		**Sample groups**			
	**Reference range**	**Standard**	**CKD**	**Nonskeletal**	**TIO**
		**(*n* = 12)**	**(*n* = 2)**	**(*n* = 3)**	**(*n* = 3)**
**Male/Female**		1/11	1/1	2/1	3/0
**Age, yr**		64 (6)	61 (9)	57 (16)	59 (8)
**Intact FGF23, pg/mL**	<30 (under hypophosphatemia)	57.1 (19.8)	20 923.8 (529.3)	52.0 (10.0)	258.5 (74.0)
**Phosphate, mg/dL**	2.5-4.5	3.6 (0.5)	4.9 (1.1)	3.5 (0.4)	2.1 (0.1)
**Albumin-corrected calcium, mg/dL**	8.8-10.1	9.3 (0.3)	10.2 (1.3)	9.0 (0.8)	8.4 (0.6)
**Alkaline phosphatase, IU/L**	38-113	88 (45)	84 (41)	82 (24)	426 (388)
**Creatinine, mg/dL**	Male: 0.65-1.07 Female: 0.46-0.79	0.58 (0.11)	8.88 (0.25)	0.72 (0.17)	0.70 (0.05)

Numerical variables are presented with mean (standard deviation).

Abbreviations: TIO, tumor-induced osteomalacia; IHC, immunohistochemistry; PIDs, phosphor-integrated dots; FGF23, fibroblast growth factor 23.

### IHC with PIDs for the quantification of FGF23 expression in normal bone

To clarify the source of excess FGF23 in the current alcoholic patient, IHC for FGF23 in the bone biopsy sample was performed. However, conventional IHC using DAB chromogen is not sensitive or quantitative enough to evaluate the expression level of FGF23 in the range of normal to suppressed values. To address this hindrance, the high sensitivity and quantifiability of IHC with PIDs attracted our attention. A schematic image of IHC with PIDs to detect FGF23 is shown in Figure S4. To assess the expression level of FGF23 in the bone from the current alcoholic case, the reference range of FGF23 expression in the bone was necessary to evaluate. For that purpose, bone samples obtained from patients with normal renal function were preliminarily subjected to IHC with PIDs.

**Figure 3:**
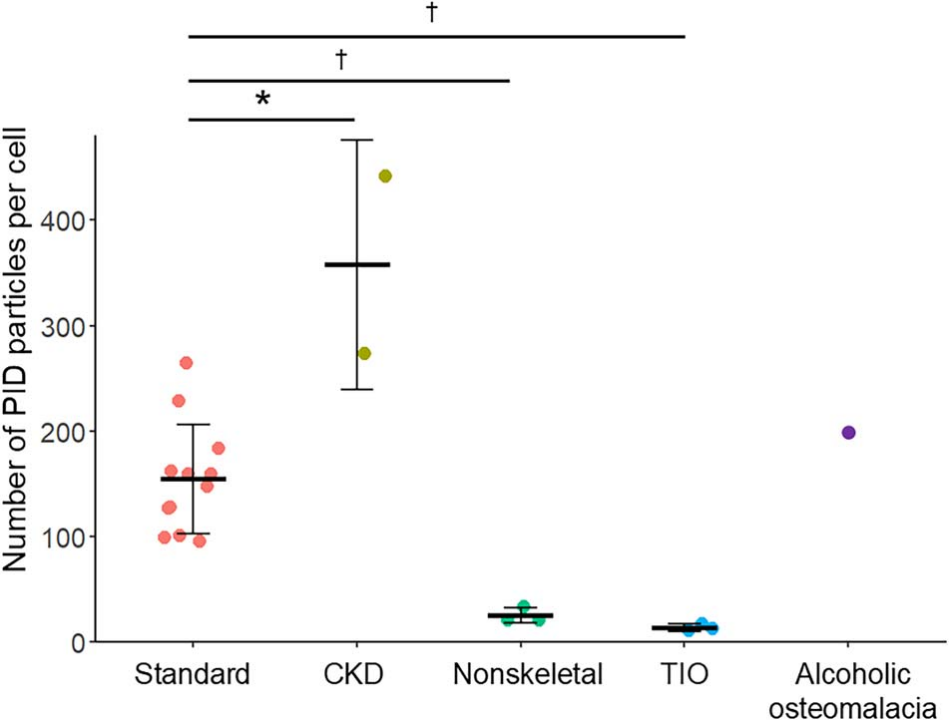
Number of PID particles in the standard group, CKD group, nonskeletal group, TIO group, and a patient with alcoholic osteomalacia. ^*^p < .05, compared with the standard group. ^†^*p* < .01, compared with the standard group. Abbreviations: TIO, tumor-induced osteomalacia; PID, phosphor-integrated dot.

Bone tissue samples were collected from 12 consecutive patients who underwent hip replacement surgery for hip osteoarthritis (standard group). The standard group consisted of 11 females (91.6%, 11/12), with a mean age of 64 yr (Table 1). All of the patients had no history of bone metabolic disorders or skeletal dysplasia and were not treated for osteoporosis. The average level of intact FGF23 (57.1 pg/mL) was within the acceptable range for normophosphatemia (Table 1). IHC with conventional DAB demonstrated patchy moderate expression of FGF23 (Figure 2A); however, IHC with PIDs was used to quantify FGF23 expression, and the average (standard deviation: SD) number of PID particles per cell among the 12 samples was 154.5 (51.6) (Figures 2A and 3).

### IHC with PIDs for the quantification of FGF23 expression in bone from patients with CKD and TIO and in nonskeletal tissues

To enable appropriate FGF23 quantification using IHC with PIDs, positive and negative control groups were used. FGF23 is prominently elevated to compensate for high serum phosphate in CKD; hence, patients with end-stage kidney disease on hemodialysis were recruited as a positive control (the “CKD group”). The CKD group was composed of the following 2 patients: a 54-yr-old male with nephrosclerosis who underwent hip replacement surgery for osteoarthritis and a 67-yr-old female with chronic glomerulonephritis who underwent hip replacement surgery for femoral neck fracture. The patients in the CKD group presented with normal-to-high levels of serum phosphate and extremely high levels of intact FGF23, as expected (Table 1).

The negative control comprised nonskeletal tissues, including the thyroid gland, lymph node, and stomach, which were obtained during surgery: thyroidectomy for a 42-yr-old female with Graves’ disease, intersphincteric resection for a 55-yr-old male with rectal cancer, and endoscopic submucosal dissection for a 74-yr-old male with early gastric cancer. These 3 patients (defined as the “nonskeletal group”) presented with serum phosphate and intact FGF23 in the reference range (Table 1).

Conventional IHC with DAB demonstrated high expression of FGF23 in the CKD group (Figure 2B), whereas only some nonspecific staining was observed in the nonskeletal group (Figure 2C). IHC with PIDs demonstrated a significantly greater number of PID particles per cell in the CKD group (mean [SD]: 357.7 [118.5], *p* = .022), and a significantly lower number of PID particles per cell was observed in the nonskeletal group (25.1 [7.3], *p* = .004) than in the standard group (Figure 3).

**Figure 4:**
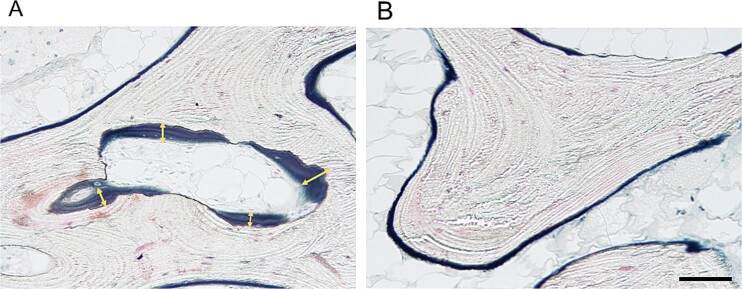
Light microscopic analysis of iliac bone biopsy from a patient with alcohol-induced hypophosphatemic osteomalacia. Villanueva bone staining demonstrated an increase in the osteoid surface (layer on the trabecular bone surface) (A and B) and an increase in the osteoid thickness (double-headed arrows) (A). The scale bar represents 100 μm.

To further confirm whether IHC with PIDs for FGF23 has the potential to differentiate FGF23-related hypophosphatemic osteomalacia due to ectopic oversecretion of FGF23 from that due to excess orthotopic FGF23 secretion from the bone, bone samples from TIO patients with concomitant hypophosphatemia were subjected to IHC with PIDs (TIO group). In the case of ectopic oversecretion of FGF23, including TIO, in which FGF23 is secreted from PMTs originating in soft or skeletal tissue, FGF23 expression in normal bone should be suppressed to compensate for hypophosphatemia. The 3 patients in the “TIO group” were all males, presenting with hypophosphatemia with inappropriately elevated intact FGF23 levels and high alkaline phosphatase activity (Table 1). The detailed information of the bone samples was as follows: the resected iliac bone for the reconstruction of the tumor removal site during surgery for PMT in the left humerus in a 67-yr-old male, the normal bone surrounding a PMT in the left acetabular bone from a 59-yr-old male with TIO, and the vertebral bone resected during surgery for an epidural PMT at the level of the ninth thoracic vertebra from a 51-yr-old man with TIO. IHC with DAB demonstrated negative expression of FGF23 in the TIO group, whereas IHC with PIDs clearly demonstrated suppression of FGF23 expression (13.6 [3.7], indicating the number of PID particles per cell), which was significantly lower than that in the standard group (*p* = .004) and comparable with that in the nonskeletal group (Figures 2D and 3).

### IHC with PIDs for the quantification of FGF23 expression in bone from patients with alcoholic osteomalacia

Finally, to investigate whether FGF23 in patients with alcoholic osteomalacia originates from orthotopic (bone) or ectopic sources (other organs, including the liver), an iliac bone biopsy sample was obtained from the current patient with alcoholic osteomalacia (4.1 yr after the first admission). One month before the bone biopsy, active vitamin D and oral phosphate supplementation were discontinued, and regular alcohol intake was continued. Under these conditions, FGF23-related hypophosphatemia was confirmed (Pi, 1.5 mg/dL; iFGF23, 113 pg/mL) (Figure 1 and Table S1). Histological observations via Villanueva bone staining demonstrated features compatible with osteomalacia, including a widened osteoid seam (yellow double arrow in Figure 4) and a reduced number of small osteoblasts. The bone histomorphometric parameters demonstrated an increase in the osteoid markers of osteoid volume (osteoid volume/bone volume: 6.0%), osteoid surface (osteoid surface/bone surface: 42.6%), and osteoid thickness (9.7 μm) (Table 2), all of which indicated preosteomalacia according to the classification in a previous report.^22^ Conventional IHC with DAB did not clearly indicate the expression status of FGF23 (Figure 2E). Subsequently, a total of 145 nuclei observed from 9 microscopic fields were analyzed using IHC with PIDs. The sum of the PID particles was 28 916, thus resulting in 199.4 PID particles per cell for this alcoholic case, which was greater than the average number of PID particles per cell in the standard group (154.5) (Figures 2E and 3).

**Table 2:** Histomorphometric analysis of the left iliac crest of a patient with alcohol-induced FGF23-related hypophosphatemic osteomalacia.

**Category**	**Item**	**Age-matched referenc**e **(SD)**	**Patient’s** **value**
**Bone volume**	Bone volume/total tissue volume, %	10.6 (2.5)	18.5
	Trabecular thickness, μm	129.5 (19.4)	138.7
**Osteoid**	Osteoid volume/total tissue volume, %		1.10
	Osteoid volume/bone volume, %	3.1 (2.0)	6.0
	Osteoid surface/bone surface, %	15.2 (4.0)	42.6
	Osteoid thickness, μm	7.4 (1.5)	9.7
	Osteoblast surface/bone surface, %		3.24
**Resorption**	Eroded surface/bone surface, %	4.1 (1.2)	2.1
	Fibrous volume/total tissue volume, %	0	0

FGF23, fibroblast growth factor 23.

## Discussion

**Figure 5:**
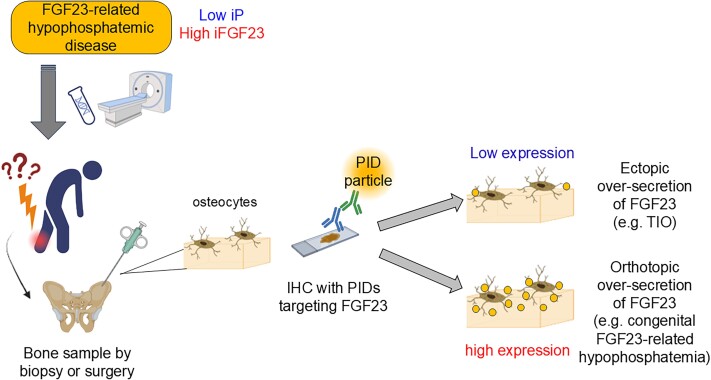
Strategy for estimating the pathophysiology of FGF23-related hypophosphatemic disease of unknown origin using IHC with PIDs. Chronic hypophosphatemia with inappropriate elevation of FGF23 levels suggests FGF23-related hypophosphatemic disease. Although clinicians first perform genetic testing and imaging studies, including ^18^F-FDG PET/CT and somatostatin receptor imaging (eg, ^68^Ga-DOTATOC PET/CT), challenges in diagnosis are noted in some cases. In such cases, IHC with PID can be preferentially utilized for differentiating its true etiology. If a low number of PID particles per cell is detected, physiological secretion from mature osteocytes is suppressed to compensate for hypophosphatemia, and ectopic oversecretion (eg, PMT in TIO or fibrous dysplasia) is considered. If a high number of PID particles per cell is observed, orthotopic oversecretion from mature osteocytes is suspected (eg, end-stage CKD or congenital FGF23-related hypophosphatemia). Abbreviations: IHC, immunohistochemistry; PIDs, phosphor-integrated dots; iP, inorganic phosphate; FGF23, fibroblast growth factor 23; ^18^F-FDG PET/CT, fluorine-18-fluorodeoxyglucose-positron emission tomography/computed tomography; ^68^Ga-DOTATOC PET/CT, gallium-68-DOTATOC positron emission tomography/computed tomography; TIO, tumor-induced osteomalacia; PMT, phosphaturic mesenchymal tumor.

This is the first study attempting to elucidate the pathophysiology of alcoholic osteomalacia with IHC with PIDs (the next-generation IHC method) with high sensitivity and quantifiability. Before the quantification of FGF23 expression in bone biopsy samples from a patient with alcoholic osteomalacia, preliminary experiments to validate the ability of IHC with PIDs to differentiate ectopic excess FGF23 secretion from orthotopic excess FGF23 secretion were conducted. The reference level of the number of PID particles per cell for FGF23 in normal bone was established to be approximately 150 by utilizing bone samples obtained from 12 patients with normal renal function. For example, when FGF23 is ectopically oversecreted from a PMT in a patient with TIO or another organ, including the liver, orthotopic FGF23 expression in the bone should be suppressed to compensate for hypophosphatemia. In contrast, when FGF23 is oversecreted from mature osteocytes (eg, in patients with congenital FGF23-related hypophosphatemia), the FGF23 expression level in the bone should be elevated (Figure 5). The utility of IHC with PIDs for the differential diagnosis of ectopic and orthotopic excess FGF23 secretion was validated, as the number of PID particles per cell in the bone from the TIO group was significantly lower than that in the reference group (25.1, *p* = .004) (Figure 3). The FGF23 expression level in the bone obtained from the current patient with alcoholic osteomalacia was rather high (number of PID particles per cell: 199) (Figure 3), thus indicating that FGF23 oversecretion in alcoholic patients was attributed to mature osteocytes (but not to the liver).

Alcoholic osteomalacia is a recently suggested entity of the acquired form of FGF23-related hypophosphatemia, and the current patient is the third reported case of such a scenario. Although a previous report from our department focused on the phenomenon in which FGF23-related hypophosphatemia turned on and off depending on the alcohol drinking status of the patients, the source of excess FGF23 secretion remained uncertain.^7^ One of the candidate organs for the ectopic source of FGF23 under these conditions was the liver. Serum C-terminal FGF23 levels were reported to be significantly greater in chronic alcoholic patients, especially those with alcoholic liver disease, than in normal controls.^8^^,^^23^ In a murine alcoholic model, Jung et al.^8^ demonstrated that hepatic FGF23 was induced under the regulation of estrogen-related receptor 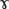, which was stimulated by alcohol-mediated activation of cannabinoid receptor type 1, although the induction of FGF23-related hypophosphatemia or reversibility of FGF23 oversecretion has not been previously described or discussed. In addition, acute and chronic liver injury has been reported to induce hepatic synthesis of FGF23 (mainly from liver macrophages or Kupffer cells) in a murine model.^24^ Two infants with biliary atresia presented with FGF23-related hypophosphatemic rickets, which improved after liver transplantation due to end-stage liver disease.^9^ Additionally, systemic inflammation increases FGF23 transcription in bone, and 1 study indicated that systemic inflammation induced by activation of the JAK1/STAT pathway led to hepatic synthesis of FGF23 through hepatic inflammation.^25–27^ Taken together, 1 possible hypothesis explaining these effects is that hepatic inflammation due to alcoholic liver disease may induce ectopic secretion of FGF23 in the liver. All 3 patients with alcoholic osteomalacia had alcoholic liver disease, and transaminases became normalized in parallel with the normalization of hypophosphatemia after abstinence. However, this study demonstrated that FGF23 oversecreted in alcoholic osteomalacia is derived from bone, although the detailed mechanism underlying the oversecretion of FGF23 in response to alcohol consumption warrants further investigation. When considering the absence of the development of FGF23-related hypophosphatemic osteomalacia among alcoholics in general, some unknown genetic or acquired factors should be involved in the development of alcoholic osteomalacia. The serum concentration of intact FGF23, which is the active form of FGF23 with phosphaturic effects, is fundamentally regulated by posttranslational modification of FGF23 by N-acetylgalactosaminyltransferase 3 (GALNAc-T3), which glycosylates the 178Thr residue in the FGF23 protein to protect it from cleavage.^28^ A study on high-phosphate diet-fed mice validated that this posttranslational modification regulation plays a pivotal role in maintaining serum phosphate levels within the optimal ranges.^29^ Patients with autosomal dominant hypophosphatemic rickets (ADHR), which is a congenital type of FGF23-related hypophosphatemia, retain variants at approximately 178Thr in the FGF23 protein, thus resulting in the secretion of cleavage-resistant FGF23.^30^^,^^31^ Among them, situations that stimulate FGF23 transcription, such as iron deficiency anemia and systemic inflammation, induce inappropriately high levels of intact FGF23 and subsequent hypophosphatemia.^32^ Hence, ADHR was first suspected to be an underlying etiology in cases of alcoholic osteomalacia, whereas whole-genome sequencing analysis of the current patient and prior 2 patients demonstrated no pathogenic or rare variants of unknown significance in *FGF23* in addition to the other 14 genes causing FGF23-related hypophosphatemic disease including 3 exploratory genes.^7^ Another form of exogenous substance-induced FGF23-related hypophosphatemia is intravenous iron preparation-induced FGF23-related hypophosphatemia, which may have a common pathophysiologic background with alcoholic osteomalacia. Notably, not all intravenous iron preparations inhibit the degradation of intact FGF23. To date, ferric carboxymaltose, ferric polymaltose, and saccharated ferric oxide have been reported to cause FGF23-related hypophosphatemia, whereas iron dextran, ferumoxytol, and ferric derisomaltose seldom cause this condition.^33–35^ Thus, some carbohydrate moieties that are incorporated into drugs to enable the gradual release of iron molecules are suspected to interfere with the posttranslational modification of FGF23 in patients with intravenous iron preparation-induced FGF23-related hypophosphatemia.^33^ Therefore, the same type of additive in alcoholic beverages may also affect phosphate regulation among patients with alcoholic osteomalacia, although the table of ingredients of the alcoholic beverages did not clearly suggest this in the present patient. Future studies that explore the genetic variants and exogenous factors related to the development of alcoholic osteomalacia using genomic and clinical data obtained from a larger number of patients with the same conditions are warranted.

This study also highlighted the high photostability, sensitivity, and quantifiability of IHC with PIDs, which is extremely superior to that of conventional IHC with DAB.^10^ IHC with PIDs is a more feasible method to quantitate FGF23 expression levels in bone tissue than RNA or protein extraction because osteocytes are scattered and deeply embedded in hard skeletal tissue; therefore, the extraction of osteocyte-derived RNA and protein is strenuous, and contamination of other types of cells in a widely variable ratio is inevitable during the procedure. Although conventional IHC with DAB to stain FGF23 is not sensitive or quantitative enough to discern normal and suppressed expression (Figure 2), new IHC technology with a wide dynamic detection range and high sensitivity is required to explore the sources of excess FGF23 in patients with alcoholic osteomalacia (Figure 5), which should be addressed by IHC with PIDs. This novel method to differentiate between ectopic and orthotopic oversecretion of FGF23 may aid in the diagnosis of TIO. Major and well-known causes of acquired FGF23-related hypophosphatemia include TIO and the administration of some intravenous iron preparations.^36^^,^^37^ In clinical practice, acquired-onset FGF23-related hypophosphatemia has been treated as TIO after excluding a history of iron infusion therapy even when a causative PMT was not identified; however, there are other etiologies for acquired-onset FGF23-related hypophosphatemia, including neurofibromatosis 1, fibrous dysplasia, and alcoholic osteomalacia.^2^^,^^36^ PMTs are not localized despite thorough functional examinations and imaging tests in approximately 30% of these “clinically suspected” cases of TIO.^1^^,^^38^ Among patients with acquired FGF23-related hypophosphatemic osteomalacia without an identified PMT, if suppressed FGF23 expression in the bone biopsy sample is confirmed by IHC with PIDs, a latent PMT is suspected, which prompts a thorough examination to detect PMT with somatostatin imaging, ^18^F-FDG PET/CT, and systemic venous sampling. Conversely, if a normal or high level of FGF23 expression is detected, germline FGF23-related hypophosphatemic disease, intravenous iron infusion-induced, alcohol-induced, or other unknown entities should be suspected, which discourages further examination with somatostatin imaging, ^18^F-FDG PET/CT, and systemic venous sampling (Figure 5). In addition, mosaic disorders with excess FGF23, such as fibrous dysplasia, McCune-Albright syndrome, and cutaneous skeletal hypophosphatemia syndrome, will also show suppressed FGF23 expression in nondysplastic bone. Genetic testing using peripheral blood exhibits less sensitivity in detecting these mosaic disorders; thus, there may be potential to utilize this new method.

There were several limitations in this study. First, liver biopsy for the described patient with alcoholic osteomalacia was not performed; therefore, direct exclusion of patients with excess hepatic expression of FGF23 was not achieved. Second, the sample size of the TIO group was small (3 patients), and it may be insufficient to conclude that the suppression of FGF23 is unexceptionally confirmed in the bone tissues of TIO patients. However, it is sometimes difficult to obtain bone samples from patients with TIO, as wide excision of PMTs in the bone is sometimes avoided due to concerns about impaired physical function, and bone samples cannot be obtained from TIO patients with PMTs in the soft tissue. Consequently, there were fewer opportunities to collect bone samples from patients with this very rare disorder. Third, the bone specimens collected from the standard, CKD, and TIO groups were not obtained through bone biopsies but were incidentally acquired during various surgeries, resulting in inconsistent sampling sites. The possibility of variation in FG23 expression based on sampling site was not taken into consideration in this study. Finally, the microscopic fields of interest were not automatically or randomly selected but were selected by a sophisticated clinical laboratory technician. Therefore, the possibility of measurement bias exists.

In conclusion, the oversecretion of FGF23 in a patient with alcoholic osteomalacia was demonstrated to be bone-derived, based on the analysis of bone samples using IHC with PIDs, which possesses much greater sensitivity and quantifiability than conventional IHC with DAB. Posttranslational modification of FGF23, which specifically occurs in mature osteocytes, plays a pivotal role in the secretion of intact FGF23; therefore, the underlying mechanisms for the deviation of this posttranslational modification should exist in patients with alcoholic osteomalacia, which warrants further research. In addition, we developed an IHC-based method to differentiate the pathophysiology of FGF23-related hypophosphatemic disease. The technique will spare costly and invasive functional tests, including PET/CT and venous sampling, to identify PMTs in acquired FGF23-related hypophosphatemic osteomalacia without the identification of PMTs.

## Supplementary Material

250107_complete_Supplementary_materials_ziaf010

## Data Availability

The data underlying this article will be shared on reasonable request to the corresponding author.
