# Supplementary material for: Excess fibroblast growth factor 23 in alcoholic osteomalacia is derived from the bone
Source: JBMR Plus. 2025 Jan 16;9(3):ziaf010. doi: 10.1093/jbmrpl/ziaf010 (PMC11831984; doi:10.1093/jbmrpl/ziaf010)
Supplement: 250107_complete_Supplementary_materials_ziaf010 [file 250107_complete_supplementary_materials_ziaf010.docx]

Supplemental Table 1. Changes in laboratory data of patients with alcohol-induced FGF23-related hypophosphatemic osteomalacia

| Laboratory results | Reference  range | Days from the first admission | | | | | | | | | |
| --- | --- | --- | --- | --- | --- | --- | --- | --- | --- | --- | --- |
|  |  | -160​ | 0​ | 15​ | 31​ | 136 | 465​ | 1004​ | 1396 | 1459​ | 1522 |
| Alcohol​ | ​ | on​ | on​ | **off​** | on​ | on | on​ | on​ | on | on​ | on |
| Inorganic phosphate |  | **off** | on | on | on | on | on | on | on | on | **off** |
| Active vitamin D |  | **off** | on | on | on | on | on | on | on | on | **off** |
| Phosphate, mg/dL​ | 2.4-4.5​ | 1.8​ | 1.4​ | **3.3**​ | 3.0​ | 2.7 | 2.0​ | 2.8​ | 2.6 | 1.6​ | 1.5 |
| Albumin-corrected calcium, mg/dL​ | 8.4-9.7​ | 9.0​ | 8.8​ | 8.6​ | 8.9​ | 9.6 | 9.0​ | 9.9​ | 10.0 | 9.5​ | 9.3 |
| Alkaline phosphatase, IU/L​ | 104-338​ | 747 | 201 | 166​ | 676 | 316 | 218​ | 189 | 157 | 145​ | 301 |
| Bone alkaline phosphatase, µg/L​ | 3.7-20.9​ | 173.9 | 160​.0 | N.E. | N.E. | N.E. | N.E. | 37.5​ | N.E. | N.E. | N.E. |
| Intact parathyroid hormone, pg/mL​ | 10-65​ | 79​ | N.E. | 111​ | 116​ | 74 | N.E.​ | 122​ | 61 | 53​ | 96 |
| 1,25-dihydroxyvitamin D, pg/mL​ | 20-60​ | 39​ | 54 | N.E. | N.E. | N.E. | N.E. | 105​ | 60 | 34​ | 24 |
| Intact FGF23, pg/mL​ | <30  (under hypophosphatemia) | 85​ | 111 | **22**​ | 205​ | N.E. | 250​ | 93​ | 115 | 108​ | 113 |
| Creatinine, mg/dL​ | 0.75-1.34​ | 0.51​ | 0.45​ | 0.53​ | 0.57​ | 0.59 | 0.71​ | 0.64​ | N.E. | 0.67​ | 0.58 |
| Aspartate aminotransferase, IU/L​ | 13-33​ | 80​ | 53​ | 30​ | 30​ | 39 | 80​ | 403​ | 97 | 104​ | 112 |
| Alanine aminotransferase, IU/L​ | 8-42​ | 58​ | 32​ | 22​ | 18​ | 26 | 42​ | 136​ | 45 | 55​ | 49 |
| γ-glutamyltransferase, IU/L​ | 13-99​ | 484​ | 254​ | 133​ | 104​ | 234 | 351​ | 498​ | 194 | 219​ | 192 |
| Iron, µg/dL​ | 40-188​ | 122​ | N.E. | N.E. | 122​ | N.E. | N.E. | 108​ | 233 | N.E. | N.E. |
| Ferritin, ng/mL​ | 23-240​ | N.E. | N.E. | N.E. | 164 | N.E. | N.E. | 191 | 172 | 134​ | 113.0 |
| TmP/GFR, mg/dL | 2.4-4.5 | 1.2 | 1.0 | 3.3 | 2.5 | 1.8 | 1.2 | 1.2 | 0.7 | 1.2 | 0.9 |
| Bone mineral density (T-score) |  | ​ | ​ | ​ | ​ |  | ​ |  |  | ​ |  |
| Lumbar spine (L2-L4)​ | ​ | 0.0​ | N.E. | N.E. | N.E. | N.E. | 1.2​ | N.E. | N.E. | N.E. | N.E. |
| Right femoral neck​ | ​ | -1.7​ | N.E. | N.E. | N.E. | N.E. | -2.0​ | N.E. | N.E. | N.E. | N.E. |
| Liver stiffness^A^, kPa |  | N.E. | N.E. | N.E. | N.E. | 8.0 | N.E. | N.E. | 15.1 | N.E. | N.E. |

^A^Liver stiffeness was measured by ultrasound-based real-time two-dimensional shear-wave elastography (2D-SWE).

FGF23: fibroblast growth factor 23; TmP/GFR: tubular maximum for phosphate reabsorption per glomerular filtration rate; N.E.: not examined

Supplemental Figure 1.


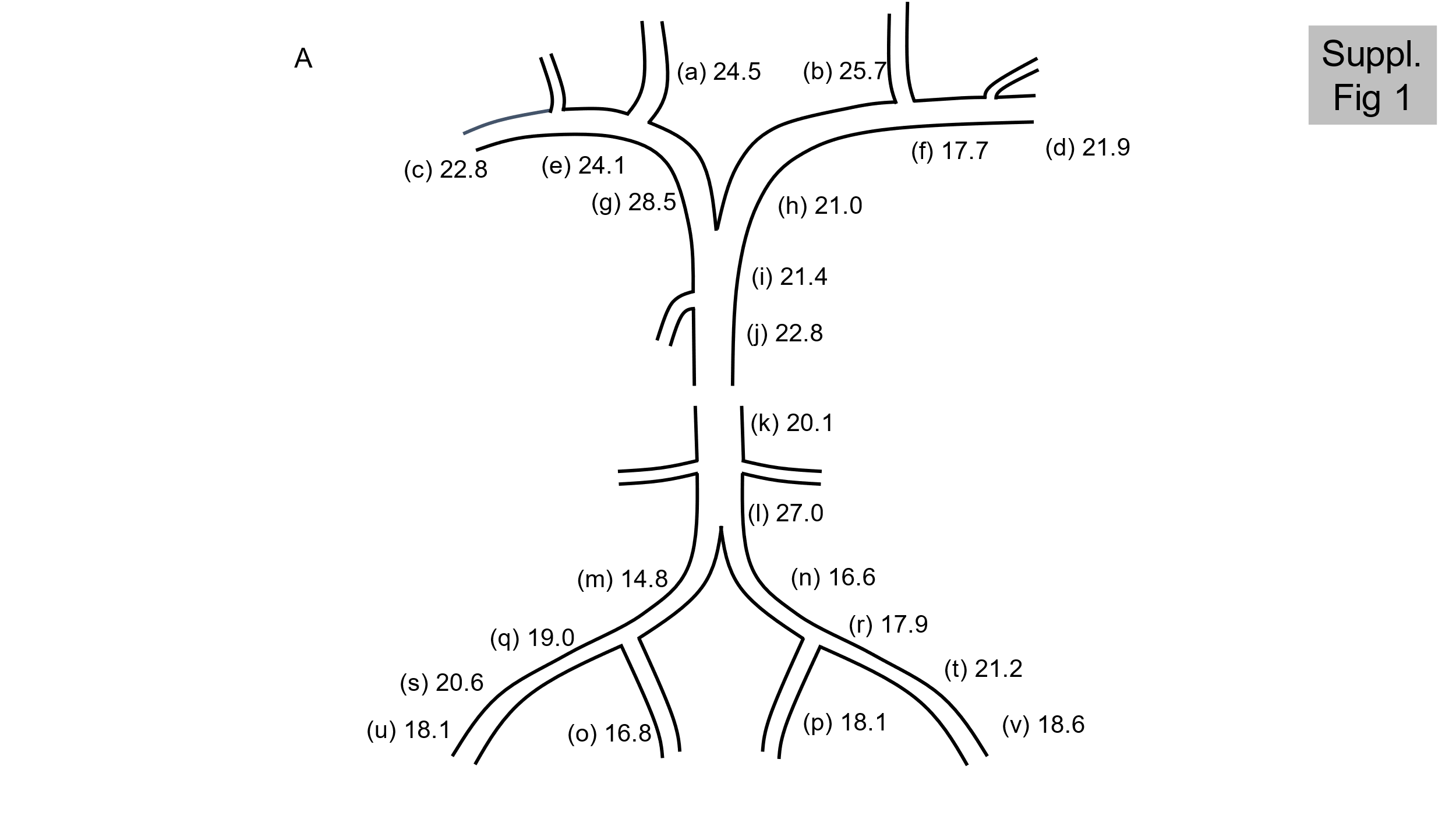

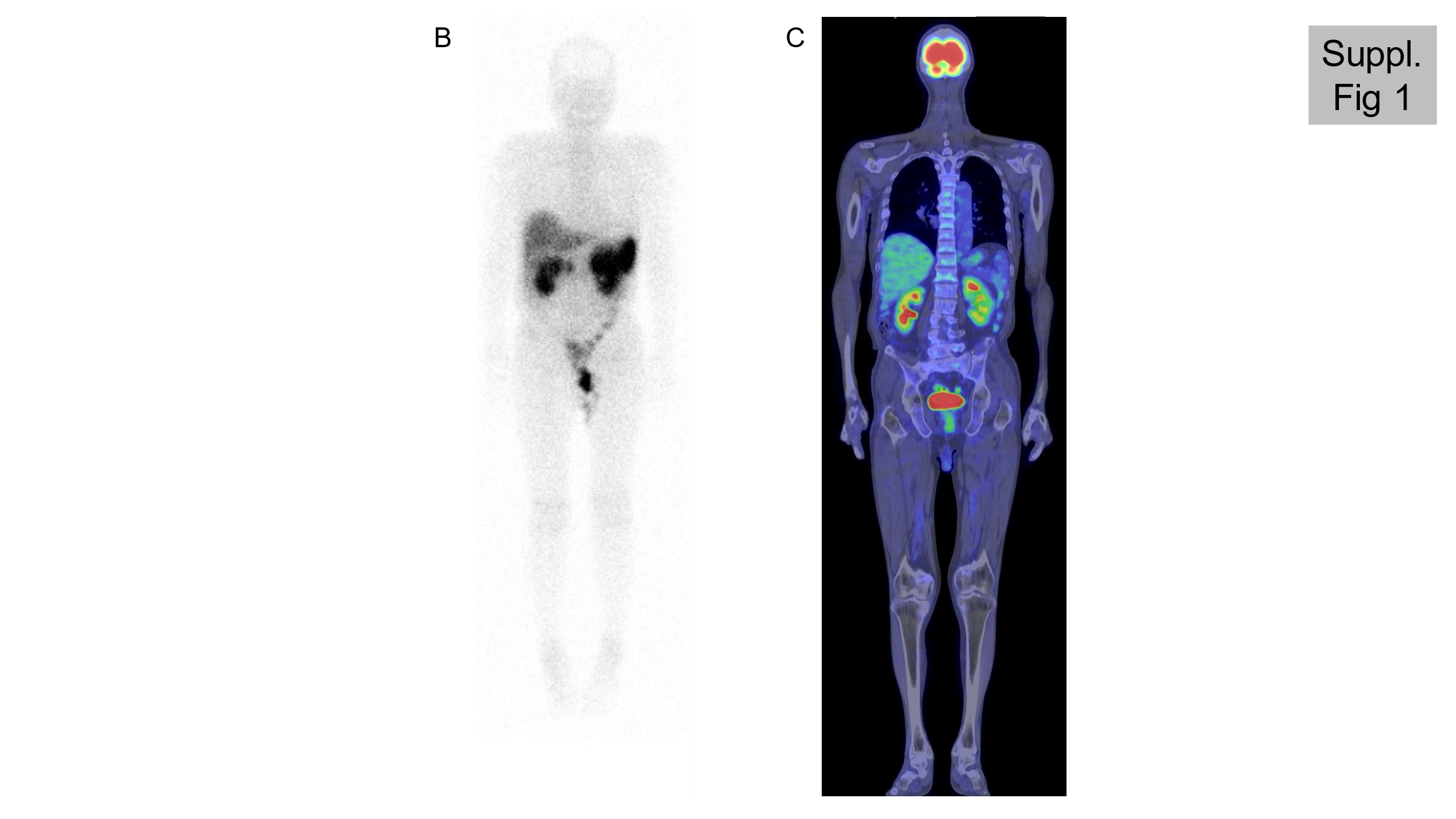


Supplemental Figure 1. The results of (A) systemic venous sampling of FGF23, (B) ^111^In-pentetreotide SPECT/CT (48 hours after the nuclide injection), and (C) ^18^F-FDG PET/CT of the patient with alcohol-induced FGF23-related hypophosphatemic osteomalacia. In (A), the values of serum intact FGF23 level (pg/mL) at the following 22 sampling points were shown: (a, b) bilateral internal jugular veins, (c, d) bilateral brachial veins, (e, f) bilateral subclavian veins, (g, h) bilateral brachiocephalic veins, (i) the distal superior vena cava, (j) the proximal superior vena cava, (k) the proximal inferior vena cava, (l) the distal inferior vena cava, (m, n) bilateral common iliac veins, (o, p) bilateral internal iliac veins, (q, r) bilateral external iliac veins, (s, t) bilateral proximal femoral veins, and (u, v) bilateral distal femoral veins.

FGF23: fibroblast growth factor 23

Supplemental Figure 2.


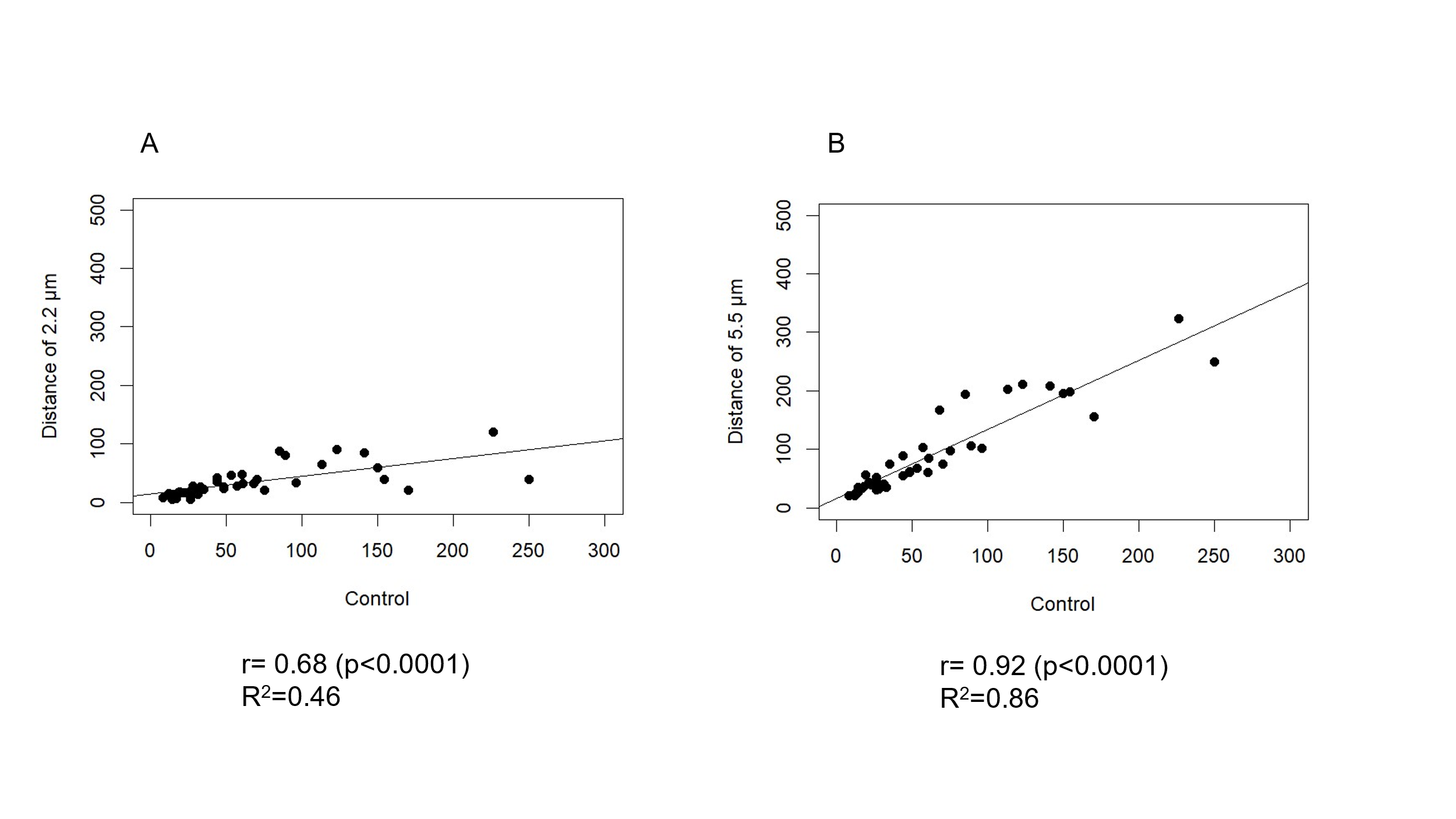


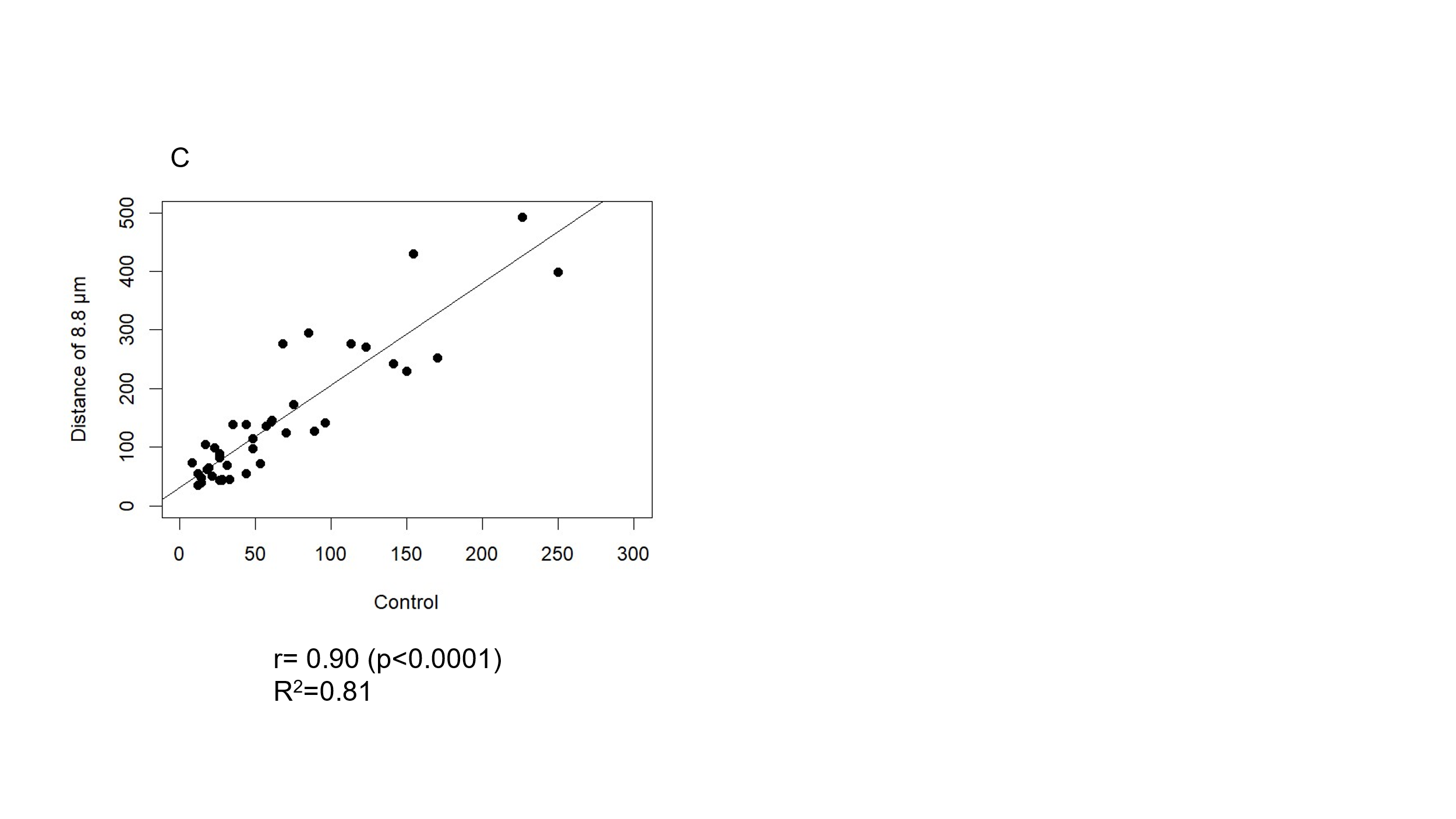


Supplemental Figure 2. Validation of the distance applied in the “nearest-neighbor method”. The scatter plots show the correlation between the value calculated by the “nearest-neighbor method” applying the distance of (A) 2.2 µm, (B) 5.5 µm, and (C) 8.8 µm and the true value (“Control” in the plots) obtained by counting PID particles per each observed cell. Pearson’s correlation efficient (“r”) with p value and coefficient of determination (“R^2^”) were calculated by R software version 4.3.2.

Supplemental Figure 3.


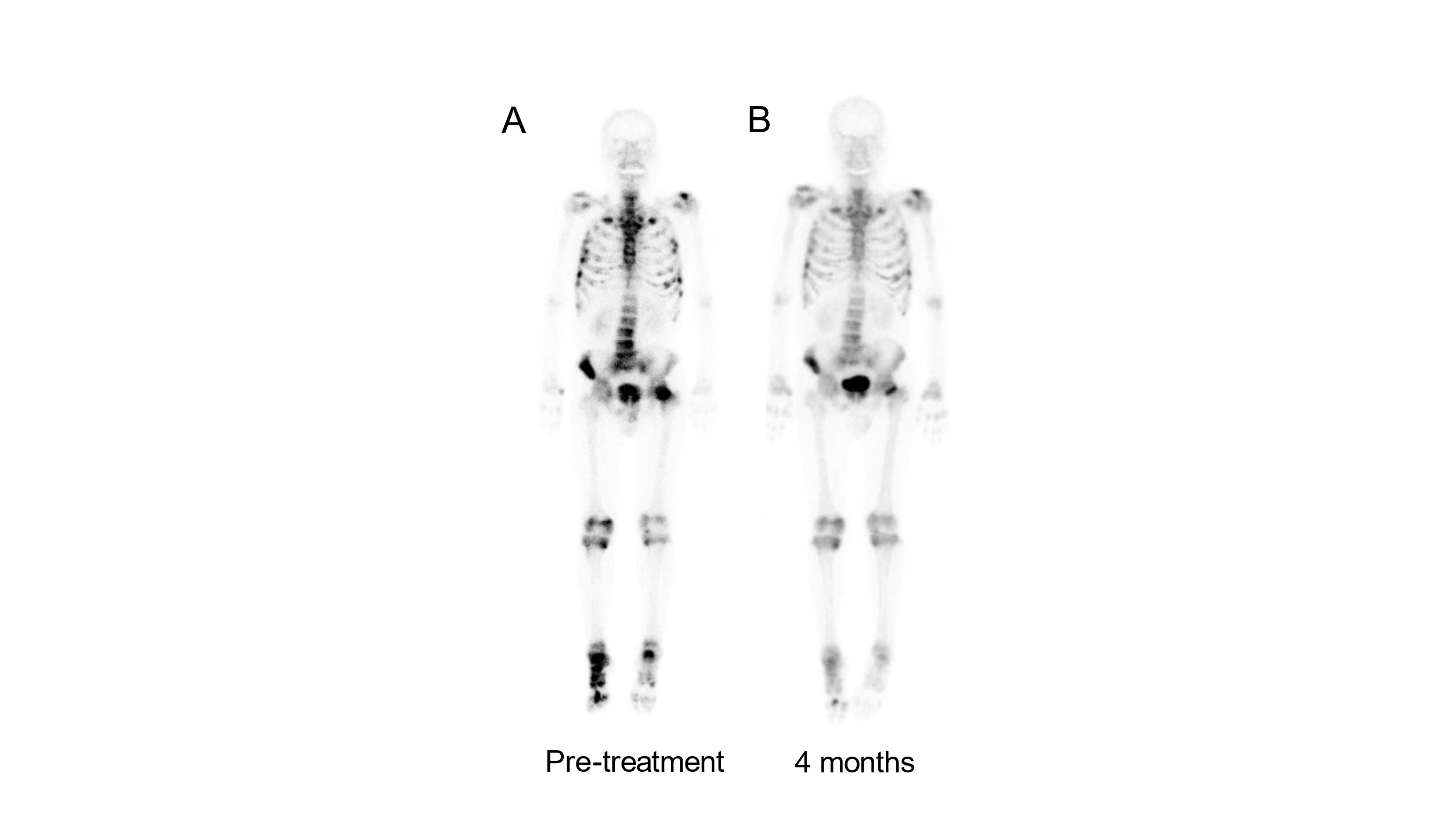


Supplemental Figure 3. Change in the ^99m^Tc-hydroxymethylene diphosphonate scintigraphy of the patient with alcoholic osteomalacia.

Anterior images at (A) pretreatment and (B) 4 months after supplementation with inorganic phosphate and active vitamin D are shown.

Supplemental Figure 4.


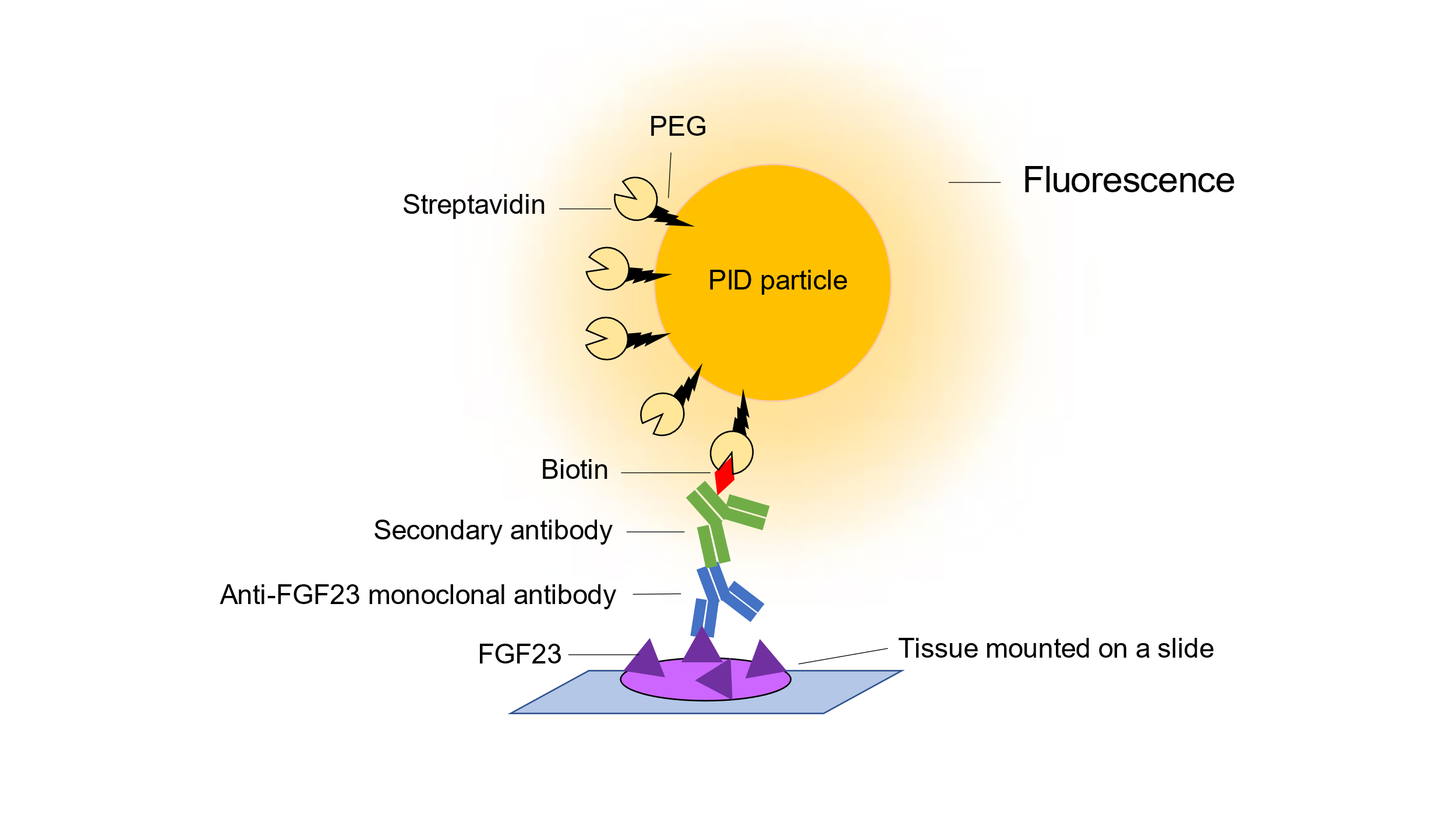


Supplemental Figure 4. Schematic of IHC with PID imaging of FGF23

A PID nanoparticle is densely packed with a fluorescent material (perylene diimide), which achieves a 100-fold greater signal than conventional fluorescent dye and high photostability due to its oxidation-resistant capsule structure. In addition, the surface of a PID particle is coated with approximately 2,460 molecules of streptavidin linked by polyethylene glycol (PEG); therefore, high reactivity is attained. The preparation of a slide and the reaction of the primary antibody (anti-FGF23 monoclonal antibody) were the same as those for conventional immunohistochemistry with DAB. After conjugation with the biotinylated secondary antibody, staining with PID particles was performed through biotin-streptavidin binding.

IHC: immunohistochemistry; PID: phosphor-integrated dot; FGF23: fibroblast growth factor 23; DAB: 3,3’-diaminobenzidine
